# Constructing prediction models for excessive daytime sleepiness by nomogram and machine learning: A large Chinese multicenter cohort study

**DOI:** 10.3389/fnagi.2022.938071

**Published:** 2022-07-29

**Authors:** Penghui Deng, Kun Xu, Xiaoxia Zhou, Yaqin Xiang, Qian Xu, Qiying Sun, Yan Li, Haiqing Yu, Xinyin Wu, Xinxiang Yan, Jifeng Guo, Beisha Tang, Zhenhua Liu

**Affiliations:** ^1^Department of Neurology, Xiangya Hospital, Central South University, Changsha, China; ^2^National Clinical Research Center for Geriatric Disorders, Xiangya Hospital, Central South University, Changsha, China; ^3^Department of Geriatrics, Xiangya Hospital, Central South University, Changsha, China; ^4^Research Institute, Hunan Kechuang Information Technology Joint-Stock Co., Ltd., Changsha, China; ^5^Department of Epidemiology and Health Statistics, Xiangya School of Public Health, Central South University, Changsha, China; ^6^Key Laboratory of Hunan Province in Neurodegenerative Disorders, Central South University, Changsha, China; ^7^Hunan Key Laboratory of Medical Genetics, Center for Medical Genetics, School of Life Sciences, Central South University, Changsha, China

**Keywords:** excessive daytime sleepiness, nomogram, Parkinson’s disease, prediction model, machine learning

## Abstract

**Objective:**

Although risk factors for excessive daytime sleepiness (EDS) have been reported, there are still few cohort-based predictive models for EDS in Parkinson’s disease (PD). This 1-year longitudinal study aimed to develop a predictive model of EDS in patients with PD using a nomogram and machine learning (ML).

**Materials and methods:**

A total of 995 patients with PD without EDS were included, and clinical data during the baseline period were recorded, which included basic information as well as motor and non-motor symptoms. One year later, the presence of EDS in this population was re-evaluated. First, the baseline characteristics of patients with PD with or without EDS were analyzed. Furthermore, a Cox proportional risk regression model and XGBoost ML were used to construct a prediction model of EDS in PD.

**Results:**

At the 1-year follow-up, EDS occurred in 260 of 995 patients with PD (26.13%). Baseline features analysis showed that EDS correlated significantly with age, age of onset (AOO), hypertension, freezing of gait (FOG). In the Cox proportional risk regression model, we included high body mass index (BMI), late AOO, low motor score on the 39-item Parkinson’s Disease Questionnaire (PDQ-39), low orientation score on the Mini-Mental State Examination (MMSE), and absence of FOG. Kaplan–Meier survival curves showed that the survival prognosis of patients with PD in the high-risk group was significantly worse than that in the low-risk group. XGBoost demonstrated that BMI, AOO, PDQ-39 motor score, MMSE orientation score, and FOG contributed to the model to different degrees, in decreasing order of importance, and the overall accuracy of the model was 71.86% after testing.

**Conclusion:**

In this study, we showed that risk factors for EDS in patients with PD include high BMI, late AOO, a low motor score of PDQ-39, low orientation score of MMSE, and lack of FOG, and their importance decreased in turn. Our model can predict EDS in PD with relative effectivity and accuracy.

## Introduction

Parkinson’s disease (PD) is a common neurodegenerative disease typically affecting individuals from middle age onward. Its main pathophysiological mechanism is the progressive degeneration of dopaminergic neurons in the substantia nigra of the midbrain and the formation of Lewy bodies in the cytoplasm of the remaining neurons in the substantia nigra. Clinically, the main manifestations are static tremor, bradykinesia, myotonia, and postural gait disorders, which are often accompanied by sleep disorders, olfactory disorders, autonomic nervous system dysfunction, cognitive disorders, and other non-motor symptoms (Bloem et al., 2021).

Sleep disturbance is a common non-motor symptom of PD. This mainly includes excessive daytime sleepiness (EDS), rapid eye movement sleep behavior disorder (RBD), restless leg syndrome (RLS), circadian rhythm sleep disorders, and sleep-disordered breathing (Zuzuarregui and During, 2020). EDS refers to the difficulty in staying awake and alert during the day, leading to pathological sleep or sleepiness, which manifests as inappropriate sleep episodes during the day. This affects the daily life of patients with PD (Suzuki, 2021). Many clinical studies have been conducted on patients with PD with EDS over the years (Maggi et al., 2021; Videovic et al., 2021; Xu Z. et al., 2021; Mehan et al., 2022). However, contributing factors for EDS in PD are controversial and need to be further researched (Bestwick et al., 2021; Xu Z. et al., 2021).

Nomograms create visual models of complex regression equations, facilitating the analysis of making the results of prediction models. Nomograms are gaining increasing attention and application in medical research and clinical practice because of their ease of interpretation and simplicity (Wu et al., 2020; Dong et al., 2021; Liu L. et al., 2021).

The severity of EDS increases with the duration of PD (Tholfsen et al., 2015; Amara et al., 2017; Xu Z. et al., 2021). EDS can seriously affect the quality of life of patients with PD, increase the financial burden to patients and their caregivers, and increase the risk of car accidents (Ueno et al., 2018; Hurt et al., 2019). Therefore, it is crucial to identify EDS and contributing factors in patients with PD and to provide prompt treatment.

Current models can predict some PD symptoms, including freezing of gait (FOG) or RBD (Xu K. et al., 2021). Nonetheless, few studies have constructed models to predict EDS in patients with PD. Our team investigated the prevalence and clinical characteristics of EDS in PD (Xiang et al., 2019) and conducted a longitudinal study with a large cohort to develop a prediction model for EDS in patients with PD. This study (1) analyzed the baseline characteristics of patients with PD with and without EDS, (2) identified risk factors for EDS at 1-year follow-up by Cox regression analysis, and (3) developed an effective and accurate prediction model for EDS in patients with PD using a nomogram and machine learning (ML).

## Materials and methods

**FIGURE 1:**
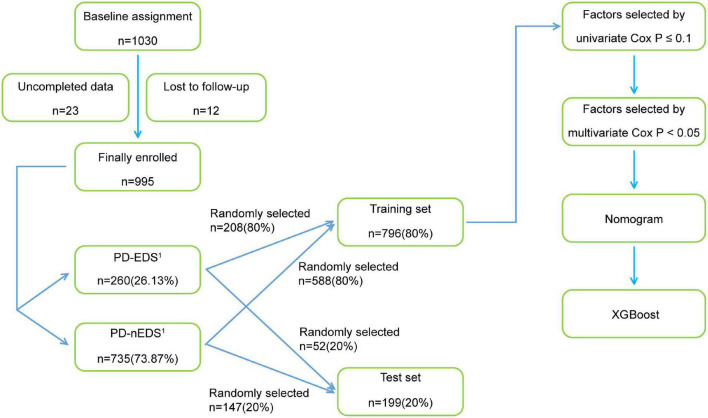
The flow chart of the study. PD-EDS^1^, patients with PD with EDS after 1 year; PD-nEDS^1^, patients with PD without EDS after 1 year; PD, Parkinson’s disease; EDS, excessive daytime sleepiness; XGBoost, eXtreme Gradient Boosting.

The study flow chart is shown in Figure 1.

### Study population

A total of 1,030 patients attending eight movement disorder centers (all 3A hospitals) in China, from January 2018 to December 2020, were recruited. None of these patients had EDS, as determined using the Epworth Sleepiness Scale (ESS; Johns, 1991). The clinical diagnostic criteria of PD used in this study were based on the International Parkinson and Movement Disorder Society (MDS) Clinical Diagnostic Criteria for PD, and the diagnosis was made by two neurologists, including clinically confirmed PD or clinically suspected PD (Postuma et al., 2015). Patients with the following were excluded: (1) chronic wasting diseases including chronic atrophic gastritis, hyperthyroidism, active tuberculosis, renal failure, and anemia with hemoglobin ≤60 g/L, (2) other diseases that involve severe drowsiness, such as obstructive sleep apnea (OSA) and hypothyroidism, (3) other diseases that may cause drowsiness confirmed by doctors to be associated with the use of medication, such as antihistamine drugs and sedatives, (4) malignant tumors or other serious systemic diseases. Based on these criteria, 995 patients with PD were included in the study. Twenty-three patients who had incomplete data and 12 patients who could not finish the interview were excluded.

This study was approved by the Ethics Committee of Central South University, Xiangya Hospital (202005124). All patients provided written informed consent before entering the study and were registered in the Multicenter Database and Collaborative Network of Parkinson’s Disease and Movement Disorders in China (PD-MDCNC).

### Independent variables and clinical assessments

The patients’ basic information and motor as well as non-motor symptoms were assessed and recorded.

Basic information included demographic characteristics, family history of PD, medical history, lifestyle, environmental factors, age of onset (AOO), disease duration, and levodopa-equivalent daily dose (LEDD; Tomlinson et al., 2015). Demographic data included sex, age, height, weight, and education level. Body mass index (BMI) was defined as weight/height^2^ (kg/m^2^), and education level was classified as primary school and below or secondary school and above. Medical history included the presence or absence of hypertension, diabetes, hyperlipidemia, heart disease, liver disease, kidney disease, respiratory disease, previous operation, tumor, stroke, traumatic brain injury (disorder of consciousness), encephalitis, epilepsy, hyperthyroidism, and mental illness. Lifestyle data included information on whether the patient smoked or consumed alcohol, coffee, or tea. Environmental factors included a history of exposure to pesticides, occupational solvents, and heavy metals, and carbon monoxide poisoning.

Motor symptoms were evaluated using the Unified Parkinson’s Disease Rating Scale (UPDRS) and the Hoehn-Yahr staging scale (H-Y). The UPDRS was used to evaluate overall motor symptoms in patients with PD (Postuma et al., 2015), and the H-Y scale was used to evaluate the severity of the disease (Hoehn and Yahr, 1967). Motor complications such as dyskinesia and wearing-off were diagnosed by clinicians, and their severity was evaluated using the Rush Dyskinesia Rating Scale (RDRS) and the 9-item End-of-dose Wearing-off Questionnaire (WOQ-9), respectively. FOG was evaluated with the New Freezing of Gait Questionnaire (NFOGQ).

The Non-motor Symptom Rating Scale (NMSS; Chaudhuri et al., 2007), Parkinson’s Disease Sleep Scale (PDSS), Parkinson Fatigue Scale (PFS), 39-item Parkinson’s Disease Questionnaire (PDQ-39), Rome III Functional Constipation Diagnostic Criteria, Mini-Mental State Examination (MMSE), Hyposmia Rating Scale (HRS), Hamilton Depression Scale (17-item version), ESS, Rapid Eye Movement Sleep Behavior Disorder Questionnaire-Hong Kong (RBDQ-HK), and Cambridge-Hopkins Questionnaire for restless leg syndrome (CH-RLSq) were used to evaluate the patients’ non-motor symptoms. The sleep quality of patients with PD was evaluated with the PDSS (Chaudhuri et al., 2002). The MMSE was used to evaluate whether patients with PD had cognitive dysfunction. Cognitive dysfunction is related to an individual’s education level. Among patients with PD with an education level of primary school and below, a total score <20 points was defined as cognitive dysfunction, while for patients with PD with an education level of middle school and above, a total score <24 was defined as cognitive impairment; lower scores denoted more severe cognitive impairment (Aarsland et al., 2001). The Hamilton Depression Scale (17-item version) was used to evaluate depression in patients with PD. A total score of >7 indicated the presence of depression (Hamilton, 1960; Montgomery and Asberg, 1979). The HRS was used to evaluate whether patients with PD had hyposmia. A total score ≤22.5 indicated the presence of hyposmia (Millar Vernetti et al., 2012). The Rome III Functional Constipation Diagnostic Criteria were used to assess whether patients with PD had constipation (Zhou et al., 2019). The PDQ-39 was used to evaluate the quality of life of patients with PD (Peto et al., 1995). The PFS was used to evaluate fatigue in patients with PD (Nillson et al., 2013). The ESS was used to evaluate EDS in patients with PD, and EDS was diagnosed at scores ≥10 (Johns, 1991). The RBDQ-HK was used to evaluate RBD in patients with PD, which could be diagnosed with a score of 18 or higher (Shen et al., 2014). The CH-RLSq was used to evaluate RLS based on the diagnostic criteria in patients with PD (Allen et al., 2009; Supplementary Table 1).

The patients’ basic information and motor and non-motor symptoms were recorded at baseline, and EDS was assessed again 1 year later. The assessment was conducted by clinicians and researchers and the results of basic information collection with questionnaires and scale assessment were recorded. Prior to the assessment, all researchers were trained to ensure an equal understanding of the scales used for clinical data collection as well as the methodology and wording used.

### Statistical analysis

#### Sample characteristics

Traditional statistical parameters were used for descriptive analyses. In the analysis of inter-group differences, for non-normal variables, particularly unordered categorical variables, the chi-square test was used. The independent-sample *t*-test and non-parametric Mann-Whitney *U* test were used for continuous variables and ordinal categorical variables, respectively. Unordered categorical variables such as FOG are presented as sample number and proportion, and continuous variables such as BMI are expressed as mean ± standard deviation. Ordinal categorical variables such as H-Y stage were plotted using distribution histograms to show the distribution of data among different grades. *P* < 0.05 was considered statistically significant.

#### The training set and the test set

A stratified random sampling method was used to divide the 995 samples into two independent sets at a ratio of 8:2, namely, a training set and a test set. The training set included 80% (796/995) of the study cohort and was initially used to establish the model. The remaining 20% (199/995) of the samples, the test set, were used along with the training set to adjust and validate the model to optimize its accuracy.

#### Cox proportional regression risk model

A Cox proportional regression risk model was used to determine the hazard ratio (HRs) of the variables affecting EDS and to construct a model to predict EDS in patients with PD. Some statistically insignificant variables were eliminated by variable preprocessing. We constructed a univariate Cox proportional risk regression model with the remaining variables in the training set. The variables with *P* ≤ 0.1 were screened into the multivariate Cox proportional risk regression model. The forward likelihood ratio (LR) method was used to screen the variables again, and the variables that were finally used to construct the prediction model were obtained. *P* < 0.05 was considered statistically significant. Survival time was set as baseline course plus 1 year. We calculated the prognostic index of each patient according to the multivariate Cox proportional risk regression model. The patients were divided into high- and low-risk groups according to the median of the prognostic index, and KM survival curves were drawn to investigate differences in survival rates according to this index. Then, we visualized the prediction model and drew line graphs, i.e., the nomograms. Finally, the model was validated. The main evaluation indexes included the C-index and time-dependent receiver operating characteristic (td-ROC) curves.

#### Machine learning

XGBoost, a ML method, is an optimized distributed Gradient Boosting library designed to be efficient, flexible, and portable. XGBoost is a massively parallel Boosting Tree tool, which is currently the fastest and best open-source Boosting Tree toolkit and is more than 10 times faster than the common toolkit (Peng et al., 2021). This study built a nomogram using a training dataset and improved the model by analyzing the test and training datasets using XGBoost. XGBoost uses these datasets to calibrate the model, to analyze the contribution of each variable to the model, and to optimize the model.

## Results

### Prevalence of excessive daytime sleepiness in the Parkinson’s disease population

A total of 995 patients with PD were included. This population had relatively complete baseline clinical data, including basic information and motor and non-motor symptoms. This population had no EDS, as assessed with the ESS, at baseline and was classified as the PD-nEDS^0^ group. The population was followed up 1 year later to reassess EDS. Our data showed that, in the PD-nEDS^0^ group, there were 260 patients (26.13%) with EDS and 735 patients (73.87%) without EDS, recorded as the PD-EDS^1^ and the PD-nEDS^1^ groups, respectively.

### Baseline clinical characteristics

In the PD-nEDS^0^ group, patients with PD were divided into PD-EDS^1^ and PD-nEDS^1^ groups according to the presence or absence of EDS in the follow-up period. Their baseline data were compared. Some statistically insignificant variables were eliminated through data preprocessing.

#### Basic information

Compared with the PD-nEDS^1^ group, the patients in the PD-EDS^1^ group were older (*p* < 0.001) and had a later AOO (*p* = 0.002). Regarding medical history, the PD-EDS^1^ group was more likely to have hypertension (*p* = 0.032). There were no statistical differences in sex, BMI, education level, family history of PD, lifestyle, environmental factors, survival time, and LEDD between the groups (Table 1).

**TABLE 1:** Comparison of basic conditions between the PD-EDS^1^ and PD-nEDS^1^ groups.

Variable	PD-nEDS^1^	PD-EDS^1^	*P*
Sex, male (n, %)	331 (45.03%)	126 (48.46%)	0.341
Age (years)	59.60 ± 10.12	62.55 ± 9.3	<0.001*
BMI	22.74 ± 3.39	22.78 ± 3.33	0.856
Education level Primary school and below (n, %)	189 (25.71%)	81 (31.15%)	0.104
Hypertension, yes (n, %)	155 (21.09%)	72 (27.69%)	0.032*
Operation history, yes (n, %)	189 (25.71%)	74 (28.46%)	0.413
Smoking, yes (n, %)	160 (21.77%)	52 (20%)	0.597
Drinking alcohol, yes (n, %)	134 (18.23%)	51 (19.62%)	0.643
AOO (years)	53.95 ± 10.61	56.29 ± 10.19	0.002*
Survival time^a^ (years)	5.61 ± 3.88	6.22 ± 4.48	0.054
LEDD	480.2 ± 394.18	510.73 ± 376.55	0.322

Survival time^a^: duration of disease plus 1 year.

*P < 0.05 (difference statistically significant).

PD-EDS^1^, patients with PD with EDS after 1 year; PD-nEDS^1^, patients with PD without EDS after 1 year; PD, Parkinson’s disease; EDS, excessive daytime sleepiness; BMI, body mass index; AOO, age of onset; LEDD, levodopa equivalent daily dose.

#### Motor symptoms

Compared with the PD-nEDS^1^ group, the UPDRS total score (*p* < 0.001), UPDRS-II score (*p* < 0.001), UPDRS-III score (*p* < 0.001), and posture gait score (*p* < 0.001) were higher in the PD-EDS^1^ group. Additionally, the PD-EDS^1^ group included more patients with FOG (*p* = 0.011). There were significant differences in the UPDRS-I score (*p* = 0.032), UPDRS-IV-B score (*p* = 0.020), UPDRS-IV-C score (*p* = 0.038), and H-Y stage (*p* < 0.001) between the groups. However, there was no significant difference in dyskinesia and wearing-off (Table 2).

**TABLE 2:** Comparison of motor symptoms between the PD-EDS^1^ and PD-nEDS^1^ groups.

Variable		PD-nEDS^1^	PD-EDS^1^	*P*
Motor subtypes	PIGD-PD (n, %)	374 (50.88%)	141 (54.23%)	0.340
	TD-PD (n, %)	299 (40.68%)	93 (35.77%)	
	Mixed PD (n, %)	62 (8.44%)	26 (10%)	
Wearing-off, yes (n, %)		150 (20.41%)	60 (23.08%)	0.377
FOG, yes (n, %)		146 (19.86%)	72 (27.69%)	0.011*
UPDRS total score (point)		35.45 ± 17.33	43.6 ± 21.74	<0.001*
UPDRS-II score (point)		9.59 ± 4.95	12.13 ± 6.24	<0.001*
UPDRS-III score (point)		22.23 ± 12.27	27.34 ± 15.21	<0.001*
Tremor score (point)		4.09 ± 3.6	4.6 ± 4.4	0.095
Posture gait score (point)		3.54 ± 2.54	4.54 ± 3.11	<0.001*
UPDRS-I score (point)		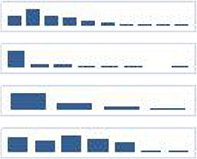	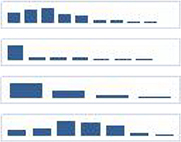	0.032*
UPDRS-IV-B score (point)				0.020*
UPDRS-IV-C score (point)				0.038*
H-Y stage				<0.001*

*P < 0.05 (difference statistically significant).

PD-EDS^1^, patients with PD with EDS after 1 year; PD-nEDS^1^, patients with PD without EDS after 1 year; PD, Parkinson’s disease; EDS, excessive daytime sleepiness; PIGD-PD, PD with postural instability/gait difficulty; TD-PD, tremor-dominant PD; FOG, freezing of gait; UPDRS, Unified Parkinson’s Disease Rating Scale; H-Y, Hoehn–Yahr.

#### Non-motor symptoms

Compared with the PD-nEDS^1^ group, NMSS-2 (*p* < 0.001), NMSS-5 (*p* = 0.004), NMSS-6 (*p* < 0.001), NMSS-7 (*p* = 0.001), NMSS-9 (*p* = 0.012) scores, and the NMSS total score (*p* < 0.001) were higher in the PD-EDS^1^ group. The PD-EDS^1^ group had a lower MMSE total score (*p* < 0.001). For PDQ-39, the PD-EDS^1^ group had higher PDQ-39 total (*p* < 0.001), motor (*p* = 0.002), activity of daily living (*p* = 0.002), cognition (*p* < 0.001), and bodily discomfort (*p* = 0.001) scores. Additionally, the PD-EDS^1^ group included more patients with RBD (*p* = 0.001) and a lower PDSS score (*p* = 0.002). There were significant differences in the scores of orientation (*p* < 0.001), attention and calculation (*p* = 0.019), recall (*p* < 0.001), and language (*p* < 0.001) domains of the MMSE between groups. However, there were no statistically significant differences in the PFS score, constipation, cognitive impairment, hyposmia, RLS, and depression between groups (Table 3).

**TABLE 3:** Comparison of non-motor symptoms between the PD-EDS^1^ and PD-nEDS^1^ groups.

Variable	PD-nEDS^1^	PD-EDS^1^	*P*
Hyposmia, yes (n, %)	263 (35.78%)	109 (41.92%)	0.086
RBD, yes (n, %)	225 (30.61%)	109 (41.92%)	0.001*
Depression, yes (n, %)	170 (23.13%)	76 (29.23%)	0.054
NMSS total score (point)	27.22 ± 20.46	35.06 ± 24.49	<0.001*
NMSS-2 (point)	6.09 ± 5.85	7.91 ± 6.21	<0.001*
NMSS-3 (point)	5.11 ± 7.85	5.48 ± 8.5	0.515
NMSS-5 (point)	2.58 ± 3.26	3.47 ± 4.47	0.004*
NMSS-6 (point)	2.89 ± 3.62	4.46 ± 4.94	<0.001*
NMSS-7 (point)	4.43 ± 5.36	5.7 ± 5.62	0.001*
NMSS-9 (point)	4.43 ± 4.69	5.36 ± 5.28	0.012*
MMSE total score (point)	27.25 ± 3	25.97 ± 4.09	<0.001*
Recall score (point)	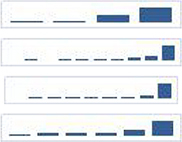	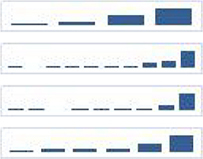	<0.001*
Language score (point)			<0.001*
Orientation score (point)			<0.001*
Attention and calculation score (point)			0.019*
PDSS score (point)	123.24 ± 22.76	117.44 ± 27.31	0.002*
PDQ-39 total score (point)	20.64 ± 19.95	27 ± 23.46	<0.001*
Motor score (point)	5.43 ± 7.95	7.53 ± 9.75	0.002*
Activities of daily living score (point)	3.8 ± 4.91	5.06 ± 5.9	0.002*
Emotional well-being score (point)	3.63 ± 5.2	4.36 ± 5.21	0.054
Stigma score (point)	2.95 ± 4.39	3.52 ± 4.79	0.092
Social support score (point)	0.25 ± 1.07	0.35 ± 1.35	0.278
Cognition score (point)	2.13 ± 2.29	2.96 ± 2.44	<0.001*
Communication score (point)	0.77 ± 1.58	1.03 ± 1.91	0.055
Bodily discomfort score (point)	1.67 ± 2.1	2.18 ± 2.18	0.001*

*P < 0.05 (difference statistically significant).

PD-EDS^1^, patients with PD with EDS after 1 year; PD-nEDS^1^, patients with PD without EDS after 1 year; PD, Parkinson’s disease; EDS, excessive daytime sleepiness; RBD, rapid eye movement sleep behavior disorder; NMSS, Non-motor Symptom Rating Scale; MMSE, Mini-Mental State Examination; PDSS, Parkinson’s Disease Sleep Scale; PDQ-39, 39-Item Parkinson’s Disease Questionnaire.

### Risk factors for excessive daytime sleepiness and construction of prediction model

Since the construction of a Cox proportional risk regression model is closely related to disease duration, we controlled for this variable and established a univariate Cox proportional risk regression model. The data showed that factors related to EDS in patients with PD included BMI, AOO, UPDRS-IV-B score, posture and gait score, hypertension, MMSE total score, orientation score, recall score, and language score, PDQ-39 total score, and motor score, wearing off, and FOG. The differences between groups were statistically significant (Table 4).

**TABLE 4:** Univariate Cox proportional risk regression model.

Variable	β-coefficient	HR (95% CI)	*P*
BMI	0.048	1 (1–1.1)	0.009*
Hypertension	0.32	1.4 (1–1.9)	0.035**
AOO	0.9	2.5 (1.8–3.4)	<0.001**
UPDRS-IV-B score	−0.12	0.89 (0.8–0.98)	0.023**
Posture gait score	−0.058	0.94 (0.9–0.99)	0.013**
H-Y stage	−0.18	0.83 (0.69–1)	0.053*
Wearing off	−0.45	0.64 (0.46–0.88)	0.0064**
FOG	−0.46	0.63 (0.46–0.88)	0.006**
MMSE total score	−0.049	0.95 (0.92–0.98)	0.0039**
Orientation score	−0.18	0.84 (0.75–0.93)	0.0012**
Recall score	−0.2	0.82 (0.72–0.94)	0.0037**
Language score	−0.1	0.9 (0.82–1)	0.039**
PDQ-39 total score	−0.0066	0.99 (0.99–1)	0.039**
Motor score	−0.022	0.98 (0.96–0.99)	0.0034**
Activities of daily living score	−0.024	0.98 (0.95–1)	0.062*
Cognition score	0.047	1 (0.99–1.1)	0.091*

*P < 0.1; **P < 0.05 (difference statistically significant).

BMI, body mass index; AOO, age of onset; UPDRS, Unified Parkinson’s Disease Rating Scale; H-Y, Hoehn-Yahr; FOG, freezing of gait; MMSE, Mini-Mental State Examination; PDQ-39, 39-Item Parkinson’s Disease Questionnaire; HR, hazard ratio; CI, confidence interval.

To avoid omitting important variables, we screened for variables with *p* ≤ 0.1 and entered these into the multifactor Cox proportional risk regression model. We set the exit value to *p* < 0.05. Finally, five variables were obtained and were included in the Cox regression equation: BMI, AOO, orientation score, motor score, and FOG. The multifactor Cox regression model is expressed as follows:


h(t,x)=h(x)0exp(0.047×BMI+0.051×AOO-0.188

×orientationscore-0.024×motorscore-0.378×FOG)

**FIGURE 2:**
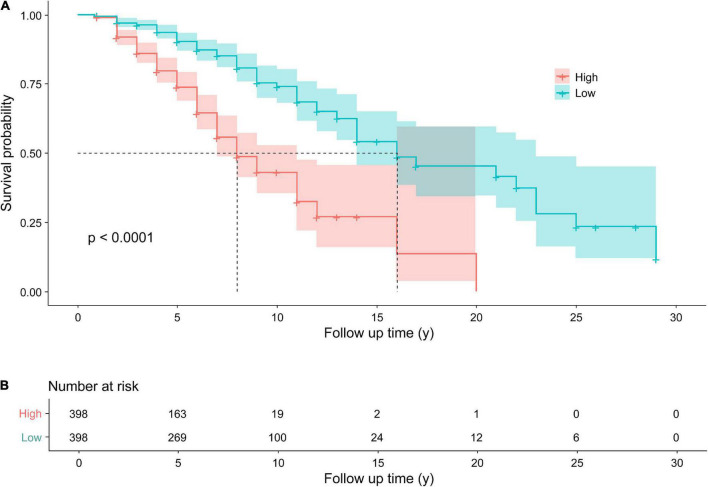
Kaplan–Meier survival curves **(A)** for patients with PD with higher and lower risks of EDS. The Kaplan–Meier curves showed a significant difference over time between patients with Parkinson’s disease with a higher risk of EDS and those with a lower risk of EDS. Follow up time (y): the time from the diagnosis of PD to the end of follow-up, namely, the course of disease at baseline plus 1 year. Number at risk **(B)** the Number of EDS in patients with PD in the high and low risk groups, respectively. PD, Parkinson’s disease; EDS, excessive daytime sleepiness.

**FIGURE 3:**
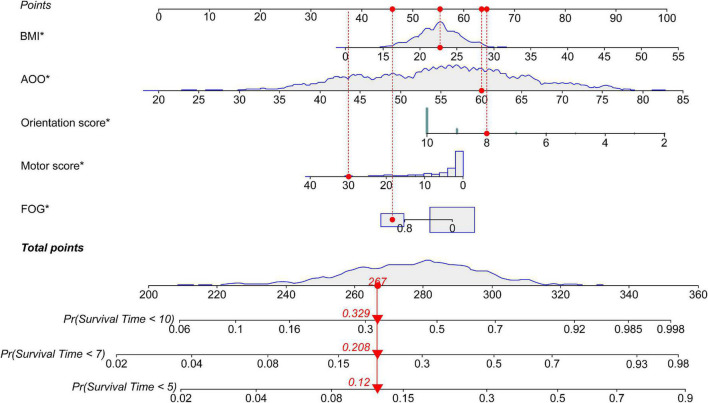
Nomogram for survival time with five variables. The nomogram showed that each factor has a score, and the total score is the sum of the scores of each factor, corresponding to the probability of EDS occurring at different survival times. Survival Time: the time from the diagnosis of PD to the end of follow-up, namely, the course of the disease at baseline plus 1 year. PD, Parkinson’s disease; EDS, excessive daytime sleepiness; BMI, body mass index; AOO, age of onset; FOG, freezing of gait. *P < 0.05.

**FIGURE 4:**
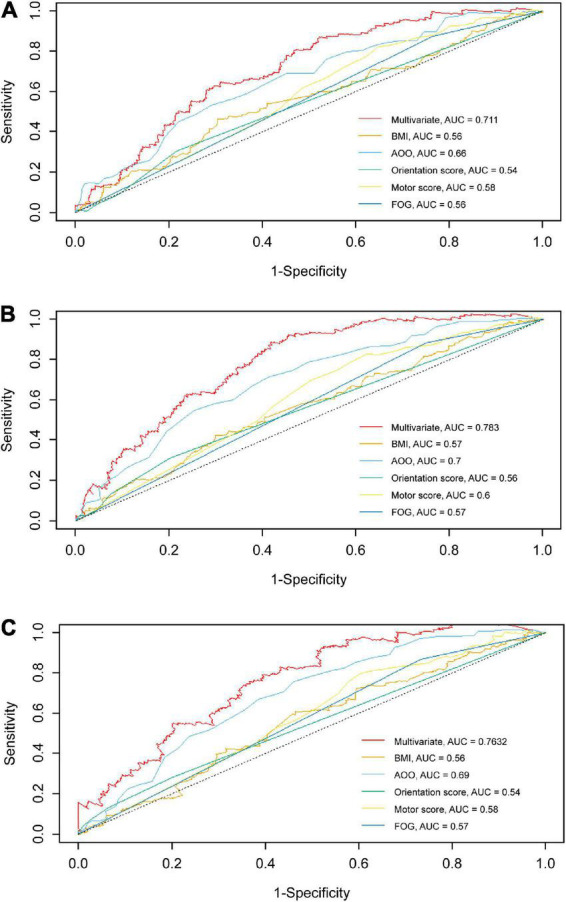
td-ROC curves of univariate and multivariate at different survival times in the training set. The td-ROC curves shows that the multivariable model has better predictive ability than other single-factor models in training set. **(A)** td-ROC of 5-year survival; **(B)** td-ROC of 7-year survival; **(C)** td-ROC of 10-year survival. td-ROC, time-dependent receiver operating characteristic; AUC, area under curve; BMI, body mass index; AOO, age of onset; FOG, freezing of gait.

According to the median of the prognosis index, the subjects were divided into the low- and high-risk groups. After plotting the Kaplan–Meier survival curve, we found that the survival prognosis of patients with PD in the high-risk group was significantly worse than that in the low-risk group (Figure 2). Additionally, through the analysis of the multifactor Cox proportional risk regression model, we found that the risk factors for EDS in patients with PD were high BMI, late AOO, low motor score, low orientation score, and lack of FOG (Table 5). Consequently, we constructed a nomogram (Figure 3). We used td-ROC curves to evaluate the predictive ability of the multivariate Cox regression model. We found that the multivariable model had better predictive ability in the training set than did single-factor models. In the training set, the 5-year area under the curve (AUC) of the Cox regression multivariable model was 0.711, the 7-year survival AUC was 0.783, and the 10-year survival AUC was 0.7632 (Figure 4). Furthermore, the C-index was 0.685.

**TABLE 5:** Multivariate Cox proportional risk regression model.

Variables	β-coefficient	HR (95% CI)	*P*
BMI	0.047	1.048 (1.009–1.089)	0.015*
AOO	0.051	1.053 (1.038–1.067)	<0.001*
Orientation score	−0.188	0.829 (0.744–0.924)	0.001*
Motor score	−0.024	0.976 (0.96–0.992)	0.004*
FOG	−0.378	0.685 (0.483–0.972)	0.034*

*P < 0.05 (difference statistically significant).

BMI, body mass index; AOO, age of onset; FOG, freezing of gait; HR, hazard ratio; CI, confidence interval.

### Performance of the prediction model based on XGBoost

Using the five variables included in the above Cox multifactor regression model equation, we adopted XGBoost to centralize the data (x) according to the minimum value, which was then scaled according to the range (maximal-minimum value). After processing, the data converged between [0, 1] and the normalized data followed a normal distribution. The equation was as follows:


x=(x-imin(x)i)/(max(x)i-min(x)i)

**FIGURE 5:**
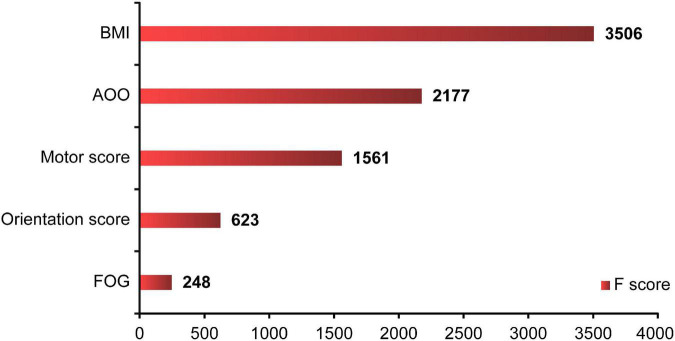
XGBoost ML analysis of the importance of each variable. The XGBoost showed that BMI, AOO, motor score, orientation score, and FOG contributed to the model in various degrees, and the importance of each variable decreased in turn. F score: the importance of each factor. XGBoost, eXtreme Gradient Boosting; ML, machine learning; BMI, body mass index; AOO, age of onset; FOG, freezing of gait.

Where model parameters included: (1) the number of trees: 1000; (2) the depth of the tree: 10; (3) the minimum weight of leaf nodes: 1; (4) penalty parameter: 0.1; (5) learning rate: 0.01; and (6) ratio of training/test set: 8:2. BMI, AOO, motor score, orientation score, and FOG contributed to the model to different degrees, and the importance of each variable decreased in that order (Figure 5). Finally, the overall accuracy of the model, which indicates the proportion of correctly predicted samples to total predicted samples, was 71.86% after testing. It showed that the model had 71.86% accuracy in predicting whether patients with PD would develop EDS.

## Discussion

No previous study has established a longitudinal EDS prediction model with large samples in patients with PD using a nomogram and ML. Our study included 995 patients with PD who were followed up for a 1-year period. We used a nomogram and the XGBoost ML method to establish an effective and relatively accurate prediction model (accuracy of 71.86%) of EDS in PD. Risk factors for EDS in patients with PD identified and included in this model were high BMI, late AOO, low motor score of PDQ-39, low orientation score of MMSE, and lack of FOG, in decreasing order of importance.

In a previous cross-sectional study of 1,221 patients with PD conducted by our team, the prevalence of EDS was 34.07% (Xiang et al., 2019). Many studies have shown that the incidence of EDS in PD increases with longer PD duration (Tholfsen et al., 2015; Amara et al., 2017; Xu Z. et al., 2021). One study found that the incidence of EDS increased from 17% to 23.3% in 2 years (Xu Z. et al., 2021). In our study, a large sample of data was used and EDS occurred 26.13% of patients with PD after 1 year of follow-up. In terms of the ESS score, a study found that the severity of EDS in patients with PD remained stable over a 10-year period (Höglund et al., 2019). Further study is required on whether the incidence of EDS in patients with PD continues to increase and if the severity of EDS worsens over a longer period of time.

The incidence of EDS in patients with PD is closely related to the type, amount, and duration of therapy for PD (Tholfsen et al., 2015). In our cohort, the type of medication was not a confounding factor and had little influence on the predictive ability of the model. The association between hypertension and increased EDS may be due to (1) an underlying sleep disorder; (2) sleep-disordered breathing; (3) higher sympathetic activity caused by sleep deprivation and increasing serum catecholamine levels; (4) changes in the neuroendocrine axis, leading to an increase in serum cortisol levels, which decrease slowly from morning to afternoon (Punjabi and Haponik, 2000).

The analysis of baseline clinical characteristics showed that patients with PD with EDS had more severe motor symptoms with respect to the UPDRS scores, H-Y stage, and FOG, suggesting that EDS is associated with severe neurodegeneration in the ascending system of the brainstem and cholinergic dysfunction in the brainstem (Muller and Bohnen, 2013). The locus coeruleus, pedunculopontine nucleus, tegmental area, and nucleus magnocellularis are important components of the ascending system of the brainstem and regulate sleep and consciousness. The impairment of these areas can lead to the development of sleep disorders, including EDS, RBD, and RLS (Saper et al., 2001; Diederich and McIntyre, 2012). In turn, patients with PD with EDS had more severe non-motor symptoms with respect to RBD and the NMSS, MMSE, PDSS, and PDQ-39 values. The impairment of executive functions, attention, and memory may be due to the degeneration of brain regions that regulate the sleep-wake cycle and corresponding changes in the levels of neurotransmitters (Braak et al., 2004; Emre et al., 2007). Moreover, the autonomic nervous system is controlled by central and peripheral neural networks, and the degeneration of these regions is associated with EDS and PD, accompanied by nocturia and nocturnal sleep disorders, such as RBD and RLS (Kingsbury et al., 2010; Weerkamp et al., 2013).

Nomograms are visual models of complex regression equations, facilitating the analysis of the results of prediction models. Nomograms are gaining increasing attention and application in medical and clinical research, especially that related to coronavirus disease and cancer, because of their ease of interpretation and simplicity (Wu et al., 2020; Dong et al., 2021; Hess, 2021).

Many clinical datasets are inaccurate, limited, cross-sectional, and lack statistical power. These problems are further complicated by the collection of different data types. ML methods learn rules directly from data and apply these rules to make predictions. In addition, these methods improve the integration of data from different models to increase statistical power (Myszczynska et al., 2020). Given its advantages and unique characteristics, ML is widely used in Internet security, data privacy, and medical, clinical, and psychological research (Orrù et al., 2020; Saura et al., 2021a,b).

XGBoost is a flexible decision tree algorithm with a simple structure and high data processing efficiency. It is suitable for large datasets with many variables. In addition, XGBoost can prevent over-fitting and has a high fault tolerance rate. As a result, redundant data do not adversely affect the accuracy of decisions. Random forest algorithms are useful to identify baseline predictors of EDS (Amara et al., 2017). However, these algorithms do not accurately analyze complex datasets and can only be applied to models with specific variables but not to other variables with inconsistent characteristics. Naive Bayes classifiers quickly and reliably find the probability distribution of features but assume that the contribution of each feature is independent, ignoring correlations between features (Orrù et al., 2020). Artificial neural networks have strong generalization and non-linear mapping capabilities, reducing the rate of errors to a certain extent. These networks are used primarily for classification and regression (Orrù et al., 2020). Thus, future studies should combine multiple ML algorithms to improve the accuracy and precision of prediction models (Mei et al., 2021).

Our results showed that patients with PD with high BMI were more likely to have EDS, and BMI had the greatest effect on the prediction efficiency of this model, which is consistent with some previous findings. In a study involving 134 patients with PD, subjective and objective sleepiness were assessed using the ESS and Multiple Sleep Latency Test, respectively (Coche De Cock et al., 2014). Patients with PD with a high BMI were found to have significant subjective and objective sleepiness (Coche De Cock et al., 2014). Additionally, in a study on patients with PD with poor nighttime sleepiness (PNS), the Pittsburgh Sleep Quality Index overall score was positively associated with BMI in patients with PNS as compared to those without (Qin et al., 2020). Our results support this viewpoint since EDS is a sleep disorder.

Furthermore, AOO may be a risk factor for EDS in patients with PD. In our prediction model, the second most important variable was AOO. The older AOO of patients with PD, the higher the risk of EDS. A study for patients with PD in Southwest China found that the prevalence of EDS in patients with late-onset PD was higher than that in patients with early-onset PD (Guo et al., 2013). Our research supports this point. Recent studies have found that patients with PD with older AOO are more likely to develop EDS within 1 year after deep brain stimulation (Jung et al., 2020). However, this is controversial, as some studies have shown that the EDS symptoms of patients with PD, including their incidence and severity, are independent of AOO (Amara et al., 2017; Liu M. et al., 2021). Therefore, future research is needed to clarify this controversy.

Many studies have found severe cognitive impairment in patients with PD. A systematic review previously showed that, compared to patients with PD without EDS, those with EDS had more serious overall cognitive dysfunction, executive dysfunction, and processing speed dysfunction, although the groups had similar language ability, memory, and visuospatial ability (Jester et al., 2020). Recently, a study showed that the ESS score of patients with PD was related to cognitive impairment including attention, working memory ability, executive function, memory, and visual space (Goldman et al., 2013). However, patients with PD with EDS are not more likely to have cognitive impairment than patients with PD without EDS (Amara et al., 2017). In the present study, we found that EDS in patients with PD was negatively correlated with the orientation score of the MMSE, which implies that the worse the orientation function of patients with PD, the higher their risk of EDS. This is consistent with some previous results. Although we evaluated patients with cognitive impairment based on their education in combination with MMSE scores, rather than on MMSE scores alone, our data suggested that overall cognitive function in patients with PD may not be significantly associated with EDS.

Previous studies have shown that patients with PD with EDS may have worse quality of life. Patients with PD with EDS have a higher total score or sub-scores in the PDQ-39 (Xiang et al., 2019; Yoo et al., 2019). However, it is worth noting that our study showed that the risk factor for EDS in PD was a lower motor score in the PDQ-39, which implies that the better the quality of life of patients with PD who exercise, the greater the risk of developing EDS in future, which is markedly different from previously reported findings. We could possibly offer an interesting explanation for this: because of the disease characteristics of patients with PD, they tend to objectively exercise less. However, in the process of diagnosis and treatment, doctors often encourage patients to exercise as much as possible because exercise helps control the symptoms and delays the development of the disease. Therefore, patients with a low motor score in the PDQ-39 and good motor function may be more inclined to exercise. However, we should also be aware that PD tends to occur in middle-aged and elderly people. When do active exercise, patients may experience rapid physical decline due to their own aging and disease, often leading to fatigue in this population, which may promote the occurrence of drowsiness and cause EDS. However, more research is needed to confirm this hypothesis.

Few studies have explored the correlation between FOG in patients with PD and EDS. Patients were divided into clinically observed and self-reported FOG in a previous study, which found a significant association with EDS in both groups (Sawada et al., 2019). Furthermore, in a 4-year longitudinal study, it was found that patients with PD with FOG had higher scores on the ESS than did our patients (PD) with a lack of FOG (Banks et al., 2019). However, patients with PD with FOG actually had a lower risk of EDS in our study, contrary to previous study results. FOG may be associated with reduced crosstalk between the frontal cortex and basal ganglia-brainstem circuits. EDS is a non-motor symptom also closely related to neurotransmitter imbalance. Therefore, we hypothesize that FOG and EDS may be related to the reduced crosstalk between these brain regions in patients with PD (Gao et al., 2020).

We built a multi-factor Cox proportional risk regression model. According to the regression coefficient (β) and hazard ratio, among the two positively correlated variables, AOO contributed the most to the prediction model. Among the three negatively correlated variables, FOG contributed the most. However, this was not consistent with the contribution of each variable to the final prediction model. A nomogram is a traditional method for preliminarily and intuitively analyzing the meanings of these variables. In fact, there was no conflict. We consider that this study adopted the method of XGBoost, which makes full use of the training and test datasets and tries to build multiple models in internally with the help of powerful computing power and then selects the best model for multiple parameter modification and adjustment to further optimize the model. The prediction model is more effective and accurate than the traditional statistical model and can better reflect the clinical value. We provide a new research method to determine the weight of each variable in the model.

### Conclusion

We studied the proportion and clinical features of 995 patients with PD without EDS who developed EDS after 1 year. We established a prediction model for EDS in patients with PD by nomogram and ML of XGBoost. Our data suggested that risk factors for EDS in patients with PD include high BMI, late AOO, low motor score of PDQ-39, low orientation score of MMSE, and lack of FOG, and their importance decreased in turn. The model we developed facilitates effective and relatively accurate prediction of EDS in patients with PD, and the overall accuracy of the model was 71.86%.

#### Theoretical implications

This study is the first to develop a longitudinal prediction model for EDS using a nomogram and ML in a large cohort with PD. This model provides a basis for the prediction of EDS in patients with PD and a new idea for the prediction of other symptoms, as well as other neurodegenerative diseases. The longitudinal analysis of large datasets increases the reliability and accuracy of the results. Our results complement other previous research results each other and can serve as the basis for understanding the pathogenesis of PD and EDS.

#### Practical implications

Clinicians can use this nomogram model to predict and to identify EDS, delaying the onset of EDS in patients with PD with early detection and treatment. Then, doctors can carry out targeted propaganda and education for patients according to the predicted results, urge patients to pay attention to their own physical condition consciously, and promote the alleviation of symptoms so that improve the quality of life of patients with PD and reduce the burden of patients and families. Furthermore, the model is visually simple, helping patients understand and accept this condition.

#### Limitations and future research

This study has limitations. First, the 1-year follow-up period may not be sufficient to assess the influence of the prediction model variables on the outcome measures, leading to representativeness bias. Second, an 11-year follow-up study showed that long naps rather than subjective EDS were a risk factor for PD (Leng et al., 2018). Therefore, future clinical studies should differentiate the groups with long naps and EDS symptoms. Third, although this result helps guide future research, higher accuracy and precision is desirable. Fourth, the ESS has limitations, such as high subjectivity, insufficient comprehensive projects, and unclear boundary of score. Furthermore, this questionnaire does not take into account individual physiological differences, medication use, presence of other sleep disorders, duration and quality of sleep, time of day for symptoms, and level of patient interest in the condition.

## Data availability statement

The data analyzed in this study is subject to the following licenses/restrictions: Data is available from the authors upon reasonable request and with permission of Multicenter Database and Collaborative Network of Parkinson’s Disease and Movement Disorders in China. Requests to access these datasets should be directed to PD-MDCNC (http://www.pd-mdcnc.com).

## Ethics statement

The studies involving human participants were reviewed and approved by the Ethics Committee of Central South University, Xiangya Hospital. The patients/participants provided their written informed consent to participate in this study.

## Author contributions

PD, BT, and JG conceived the study. PD, YL, and HY analyzed and interpreted the data. PD edited the manuscript. PD, KX, XW, JG, BT, and ZL discussed the results. PD, KX, BT, and ZL discussed and revised the manuscript. PD, KX, XZ, YX, QX, QS, XY, JG, BT, and ZL provided clinical data. All authors made some contribution to the manuscript and approved the submitted version for publication.
