# Supplementary material for: Constructing prediction models for excessive daytime sleepiness by nomogram and machine learning: A large Chinese multicenter cohort study
Source: Front Aging Neurosci. 2022 Jul 29;14:938071. doi: 10.3389/fnagi.2022.938071 (PMC9372350; doi:10.3389/fnagi.2022.938071)
Supplement: Supplementary file 1 [file Table_1.docx]

Supplementary Material

# Supplementary Tables

**Supplementary Table 1.** Measures and characteristics of independent variables of non-motor symptom

| Independent variables | Measures | Characteristics |
| --- | --- | --- |
| Non-motor symptom | Non-Motor Symptom Rating Scale (NMSS) | + |
| Sleep quality | Parkinson’s Disease Sleep Scale (PDSS) | - |
| Fatigue | Parkinson Fatigue Scale (PFS) | + |
| Quality of life | 39-item Parkinson’s Disease Questionnaire (PDQ-39) | + |
| Constipation | Rome III Functional Constipation Diagnostic Criteria | # |
| Cognitive dysfunction | Mini-Mental State Examination (MMSE) | * |
| Hyposmia | Hyposmia Rating Scale (HRS) | Score ≤ 22.5 |
| Depression | Hamilton Depression Scale (17-item version) | Score > 7 |
| EDS | Epworth Sleepiness Scale (ESS) | Score ≥ 10 |
| RBD | Rapid Eye Movement Sleep Behavior Disorder Questionnaire‒Hong Kong (RBDQ-HK) | Score ≥ 18 |
| RLS | Cambridge‒Hopkins Questionnaire for restless leg syndrome (CH-RLSq) | ^ |

+: the higher the score, the more severe the symptoms; -: the lower the score, the more severe the symptoms; #: it depends on medical history; *: among patients with PD with an education level of primary school and below, a total score < 20 points was defined as cognitive dysfunction, while for patients with PD with an education level of middle school and above, a total score < 24 was defined as cognitive impairment, and the lower the score, the more severe the symptoms; ^: it based on the diagnostic criteria of CH-RLSq. PD, Parkinson’s disease; EDS, excessive daytime sleepiness; RBD, rapid eye movement sleep behavior disorder; RLS, restless leg syndrome.
